# Ultrasound Parameters for Assessment and Rehabilitation Monitoring of Post-Stroke Dysphagia: A Narrative Review

**DOI:** 10.3390/life16101727

**Published:** 2026-10-15

**Authors:** Ji-Youn Kim, Haneul Lee

**Affiliations:** 1 Department of Dental Hygiene, Gachon University, 191, Hambangmoe-ro, Yeonsu-gu, Incheon 21936, Republic of Korea; hoho6434@gachon.ac.kr; 2 Department of Physical Therapy, Gachon University, 191, Hambangmoe-ro, Yeonsu-gu, Incheon 21936, Republic of Korea

**Keywords:** post-stroke dysphagia, ultrasonography, swallowing, rehabilitation, ultrasound parameters

## Abstract

Post-stroke dysphagia is a common complication of stroke. Quantitative ultrasonography has emerged as a noninvasive, radiation-free, and repeatable approach for evaluating swallowing-related structures and movements. This narrative review summarizes quantitative ultrasound parameters used for swallowing assessment and rehabilitation monitoring in patients with stroke. Ultrasound-derived measurements were categorized according to muscle quantity and morphology, muscle quality, spatial/kinematic function, and temporal/velocity function. Studies have demonstrated alterations in swallowing-related muscle morphology; reductions in the movement of the tongue, suprahyoid musculature, hyoid bone, and hyolaryngeal structures; and changes in movement duration and velocity. Several dynamic measures have been associated with the severity of dysphagia and conventional swallowing assessments. Ultrasonography has also been used longitudinally to monitor rehabilitation-related changes in muscle morphology and swallowing kinematics across different therapeutic interventions. However, improvements in swallowing function were not consistently accompanied by changes in individual ultrasound parameters. Dynamic ultrasound parameters have the potential to characterize swallowing impairment and monitor rehabilitation-related changes, whereas quantitative ultrasonography may provide a complementary approach to repeated bedside assessments. Further studies using standardized acquisition protocols, clearly defined parameters, and validation against established swallowing assessments are required to support broader clinical applications.

## 1. Introduction

Post-stroke dysphagia is a common and clinically important complication of stroke [1,2]. Although swallowing function improves spontaneously in many patients during the early phase of recovery, dysphagia may persist and continue to affect their nutritional intake, respiratory health, and participation in rehabilitation [3,4]. These clinical consequences extend beyond difficulty eating and drinking. Impaired bolus control and airway protection increase the risk of aspiration and stroke-associated pneumonia, while reduced oral intake may contribute to malnutrition and dehydration [5]. Post-stroke dysphagia is also associated with prolonged hospitalization, greater dependency, poor functional recovery, and increased mortality [6]. Early identification and continuous monitoring are essential for acute management and subsequent rehabilitation.

Traditionally, swallowing has been evaluated primarily in terms of functional performance. Clinical and instrumental assessments focus on whether a bolus is transported safely and efficiently through the oropharynx, whether airway protection is maintained, and whether penetration or aspiration occurs [7,8]. Videofluoroscopic swallowing study (VFSS) and fiberoptic endoscopic evaluation of swallowing (FEES) are the principal instrumental methods for evaluating swallowing physiology and airway safety [2]. These assessments are indispensable for identifying aspiration, pharyngeal residues, and abnormalities in bolus transit. Although these assessments provide detailed information on swallowing physiology and bolus flow, they provide limited direct information on the morphology, quality, and contractile behavior of the muscles that generate these movements [9,10]. Consequently, patients with similar functional findings may have different underlying muscular impairments that cannot be fully characterized by assessments focused primarily on bolus flow and airway safety. This distinction is particularly relevant after stroke, as swallowing impairment results from the disruption of neural control and coordinated activation of multiple oropharyngeal muscles.

Swallowing is a complex sensorimotor process controlled by distributed cortical, subcortical, brainstem, and peripheral neural pathways [11,12]. Cortical and subcortical regions provide supramedullary control of swallowing, while brainstem networks integrate descending and peripheral sensory inputs to coordinate the sequential motor activity required for swallowing [12]. Sensory information from the oral cavity, tongue, pharynx, and larynx is conveyed mainly through the trigeminal (V), glossopharyngeal (IX), and vagus (X) nerves, with taste-related input also transmitted through the facial nerve (VII); these afferent signals contribute to the initiation and modulation of swallowing [13]. Motor output through cranial nerves V, VII, IX, X, and XII coordinates the activation of muscles involved in movements of the jaw, lips, palate, pharynx, larynx, and tongue [12]. Consequently, stroke involving cortical, subcortical, or brainstem swallowing networks may disrupt the neural control of swallowing and result in impaired coordination of swallowing-related movements [11,12].

The tongue generates and controls pressure for bolus formation and propulsion, whereas the suprahyoid muscles contribute to hyolaryngeal elevation and anterior displacement required for airway protection and opening of the upper esophageal sphincter [11,12]. Stroke-related impairments in the timing, strength, and coordination of these muscles may lead to inefficient bolus transport, pharyngeal residue, and aspiration. Stroke may also result in asymmetric or weakened tongue and palatal function, which may further contribute to swallowing impairment [14,15]. Reduced oral intake, physical inactivity, and prolonged use of modified diets or tube feeding may further contribute to disuse-related deterioration of the swallowing muscles, particularly in older patients with coexisting sarcopenia [16,17]. Therefore, the assessment of post-stroke dysphagia should extend beyond the observable outcome of swallowing to include the muscular structures and contractions responsible for that outcome.

Ultrasound provides a practical means of examining the underlying muscular components. As a noninvasive imaging modality that does not involve ionizing radiation, it enables the repeated assessment of swallowing-related muscles and structures [18,19,20]. Importantly, ultrasonography can characterize both muscle morphology and swallowing-related movements, thereby complementing functional swallowing assessments with quantitative information on the muscular and kinematic mechanisms underlying dysphagia [10,20,21].

Previous reviews have described the broader use of ultrasound for swallowing assessment across heterogeneous populations or in neurogenic dysphagia more generally [9,10,20,22]. These reviews have established the feasibility of ultrasound for examining swallowing-related structures and movements; however, they have not comprehensively integrated stroke-specific evidence across morphological and dynamic ultrasound parameters or examined their potential roles in rehabilitation monitoring. Accordingly, this narrative review focuses on ultrasound-derived parameters of the tongue and suprahyoid muscles, together with the associated hyoid and hyolaryngeal movements, in individuals with stroke. Specifically, it examines: (1) which anatomical structures, image acquisition approaches, and static or dynamic ultrasound parameters have been evaluated in stroke; (2) how these parameters differ between individuals with and without post-stroke dysphagia and are related to swallowing impairment or clinical outcomes; and (3) whether they are responsive to treatment-related changes and may support rehabilitation monitoring. By addressing these areas, this review provides a stroke-specific integration of morphological and dynamic ultrasound parameters, with particular emphasis on their potential role in rehabilitation monitoring.

## 2. Literature Search and Review Scope

A literature search was conducted using Embase and Ovid MEDLINE to identify relevant studies published through May 2026. The search terms included a combination of those related to ultrasonography, swallowing-related muscles and structures, ultrasound-derived parameters, dysphagia, and rehabilitation. No language restrictions were applied, and only original research articles evaluating the tongue, suprahyoid muscles, and hyoid and hyolaryngeal movements using ultrasonography in individuals with stroke were eligible for inclusion. The titles, abstracts, and full texts of potentially relevant articles were independently reviewed by two reviewers (J.-Y. Kim and H. Lee). Any disagreements regarding study inclusion were resolved through discussion. The identified evidence was synthesized narratively, with emphasis on ultrasound parameters, acquisition approaches, clinical associations, and rehabilitation monitoring. Fourteen original studies were included in the narrative synthesis.

## 3. Ultrasound Assessment of Swallowing in Stroke

### 3.1. Capabilities and Practical Advantages of Ultrasound

Ultrasound enables direct visualization of the muscles and anatomical structures involved in swallowing without exposing patients to ionizing radiation. Depending on the imaging approach and anatomical window, it can be used to examine the tongue, geniohyoid, mylohyoid, digastric muscles, hyoid bone, larynx, and other suprahyoid structures [20,23]. As these structures differ in anatomical location, orientation, and contribution to swallowing, the transducer position and imaging plane must be selected according to the target structure and specific clinical or research questions. This flexibility allows ultrasound to provide muscle- and structure-specific information using a single imaging modality.

A major advantage of ultrasound is its ability to evaluate both structural and dynamic characteristics of the swallowing muscles. Static B-mode imaging can be used to quantify muscle thickness, cross-sectional area, and echo intensity, thereby providing information on muscle quantity and quality [22,24]. Dynamic imaging can capture changes in muscle thickness during contraction, tongue movement, hyoid and laryngeal displacement, contraction duration, and other temporal or kinematic features of swallowing [10,20,23]. These two approaches are complementary: static measures characterize the condition of the muscle at rest, whereas dynamic measures reflect how the muscle or related structures behave during a swallowing task. This distinction may be particularly relevant after a stroke, in which swallowing impairment can involve altered neural control, timing, and coordination, in addition to structural changes.

Ultrasound offers several practical advantages in the assessment of patients with stroke. It is noninvasive, portable, relatively inexpensive, and suitable for repeated examinations with minimal interference with the swallowing process [19,20]. Measurements can be obtained at the bedside, which is particularly useful for patients with reduced mobility, impaired consciousness, medical instability, and limited access to radiological facilities [9,25]. The absence of ionizing radiation further facilitates serial assessments during recovery or rehabilitation [20]. These characteristics make ultrasound particularly suitable for longitudinal evaluation in which changes in swallowing muscle morphology or movement can be monitored over time.

Nevertheless, information obtained by ultrasound depends on the acoustic accessibility of the target structure, examiner expertise, probe placement, and measurement protocol used [26]. Also, ultrasound does not provide comprehensive visualization of bolus flow and airway invasion as provided by established instrumental assessments such as VFSS or FEES [19]. Therefore, its principal value lies in providing quantitative information about the muscular and kinematic mechanisms underlying swallowing performance rather than independently establishing the overall safety of swallowing.

### 3.2. Ultrasound Parameters Used for Swallowing Assessment

#### 3.2.1. Muscle Quantity and Morphology

Commonly used ultrasound parameters include measures of muscle quantity and morphology, which are primarily obtained using B-mode ultrasonography. Muscle thickness is a linear measure of the size of a target muscle or muscle region [22,23]. Detailed measurement methods vary according to the anatomical characteristics of the target structure, including the imaging plane, probe position, and anatomical landmarks used for measurement [20,23]. Muscle thickness is the simplest and most widely adopted parameter because it is straightforward, reproducible, and suitable for repeated bedside examinations [20,22,23]. Thickness measurements were performed on the tongue and suprahyoid muscles, including the mylohyoid and geniohyoid [27,28,29].

Cross-sectional area (CSA) provides a two-dimensional measure of muscle size obtained from cross-sectional ultrasound images [22,23]. The CSA has been applied to swallowing-related muscles, such as the geniohyoid, anterior belly of the digastric muscle, and tongue, to characterize muscle quantity and regional morphology [27,30]. Collectively, these measurements describe the structural characteristics of swallowing muscles and provide objective indicators of muscle quantity.

#### 3.2.2. Muscle Quality

Muscle quality provides complementary information beyond muscle morphology. Ultrasound can also evaluate muscle quality by characterizing tissue composition, with echo intensity and echogenicity representing commonly used parameters [22,23]. These measurements may reflect pathological changes such as fatty infiltration, fibrosis, or alterations in muscle architecture that influence contractile performance independently of muscle size [31,32,33]. Although these parameters have been increasingly investigated in musculoskeletal and neuromuscular disorders, their application to swallowing assessment remains relatively limited [19,23]. Nevertheless, muscle quality assessment represents a promising direction for future swallowing research because it may complement conventional morphological measurements.

#### 3.2.3. Dynamic and Functional Parameters

Recent advances in swallowing ultrasonography have shifted the focus from static anatomical assessment toward dynamic evaluation of swallowing biomechanics [10,20,22]. Dynamic ultrasound parameters quantify real-time changes in swallowing-related muscles and anatomical structures and include changes in tongue thickness during swallowing [34] and suprahyoid muscle contraction-related measures [35,36], as well as movement-related measures such as tongue and geniohyoid displacement [37], suprahyoid muscle displacement [35,36], hyoid displacement [34,37,38], and hyoid–larynx approximation distance and approximation ratio [25,37,39]. Additional dynamic measures include tongue and geniohyoid movement speed [38] and temporal parameters such as movement duration [37], peak-to-peak time, and total duration [35,36]. These parameters can be obtained using B-mode and M-mode ultrasonography [20,34,35,36,37]. As ultrasonography technology has evolved, composite indices integrating movement amplitude and temporal characteristics have also been introduced, providing a more comprehensive description of swallowing biomechanics [35,36].

Because swallowing depends on the precisely coordinated activation of multiple bilateral muscles and anatomical structures, bilateral and multi-structural measurements represent potential extensions of dynamic ultrasound assessment [10,20]. Bilateral measurements may provide information on side-to-side differences in muscle morphology or movement, particularly in patients with unilateral neurological lesions. However, standardized symmetry indices and direct ultrasonographic measures of temporal or spatial coordination among multiple swallowing structures have not yet been established [10,20,22].

### 3.3. Comparison with Conventional Swallowing Assessments

Conventional swallowing assessments differ from ultrasound in both their clinical purpose and the type of information they provide. In patients with acute stroke, early dysphagia screening is recommended before oral intake to identify patients with dysphagia or aspiration risk and to determine the need for further swallowing assessment [2,40]. Patients who fail the initial screening subsequently undergo a more comprehensive dysphagia assessment, typically based on a clinical swallow examination and/or instrumental assessment using VFSS or FEES [2]. Clinical swallow examination is accessible and suitable for bedside use but cannot directly visualize bolus flow, aspiration, or the swallowing musculature. VFSS and FEES provide more detailed instrumental evaluation and remain central to the assessment of post-stroke dysphagia. VFSS visualizes bolus transit and swallowing biomechanics across the oral and pharyngeal phases and can identify penetration, aspiration, residue, and abnormalities in hyolaryngeal movement. FEES directly visualizes pharyngeal and laryngeal structures, secretions, residue, penetration, and aspiration before and after the pharyngeal phase of swallowing and can be performed repeatedly at the bedside [2].

Despite their clinical importance, VFSS and FEES are not designed primarily to characterize the morphology or tissue composition of individual swallowing muscles. VFSS exposes patients to ionizing radiation and requires radiological equipment, which may restrict frequent serial assessment. FEES does not involve radiation and is well suited to bedside evaluation, but visualization is temporarily obscured during pharyngeal constriction by the characteristic white-out period. More importantly, neither examination directly quantifies features such as tongue or suprahyoid muscle thickness, cross-sectional area, or echo intensity. Ultrasound can therefore add information about the muscular structures producing the swallowing movements observed during VFSS or FEES [19,20].

Tongue pressure measurement and surface electromyography provide additional information about swallowing-related muscle performance. Tongue pressure quantifies the force generated by the tongue against the palate and is useful for identifying reduced lingual strength. However, it does not indicate whether reduced pressure is associated with altered muscle size, tissue quality, contraction pattern, or movement. Surface electromyography records the electrical activity of superficial or submental muscle groups and can provide information on the onset, duration, and relative magnitude of muscle activation. Its ability to identify the contribution of individual deep muscles is limited, however, because signals may represent the combined activity of several adjacent muscles and may be affected by electrode placement and crosstalk. Ultrasound does not measure muscle force or electrical activity directly but visualizes the corresponding structural deformation and movement. Consequently, these measures should be viewed as complementary rather than interchangeable.

The distinction among these methods can therefore be understood in terms of the clinical question being addressed. VFSS and FEES primarily determine what happens to the bolus and whether swallowing is safe; clinical assessment identifies observable signs and risks; tongue pressure estimates force-generating capacity; surface electromyography reflects muscle activation; and ultrasound characterizes muscle morphology and swallowing-related movement. Ultrasound should therefore not be positioned as a replacement for VFSS or FEES. Instead, it provides an additional muscle-centered layer of information that may help explain the structural and dynamic impairments underlying abnormal swallowing performance and facilitate repeated monitoring during post-stroke recovery and rehabilitation [10,22].

## 4. Ultrasound Findings in Patients with Stroke

The findings from the studies included in this review indicate that post-stroke dysphagia is associated with abnormalities in both static muscle morphology and dynamic swallowing biomechanics. However, the consistency of these findings varies across ultrasound parameter categories (Table 1).

### 4.1. Alterations in Muscle Morphology and Quantity

Muscle thickness has frequently been evaluated as a morphological parameter in stroke-related swallowing ultrasound studies. Alterations in muscle thickness have been reported in several swallowing-related muscles. Tongue thickness does not significantly differ between patients with post-stroke dysphagia and healthy controls [41], whereas mylohyoid thickness is lower in patients with stroke and dysphagia than in those without dysphagia [27]. Reduced thickness of the suprahyoid muscle complex, comprising the geniohyoid, mylohyoid, and anterior belly of the digastric muscles, has also been observed in patients with more severe dysphagia [35]. However, a reduction in muscle thickness has not been consistently observed across all studies. For instance, the sagittal tongue base thickness, representing the posterior tongue region, was greater in patients with acute ischemic stroke than in healthy controls and was not significantly associated with swallowing screening results [42]. These findings indicate that alterations in muscle thickness after a stroke may vary according to the anatomical structure examined, stroke stage, and measurement approach. CSA has been investigated less frequently than muscle thickness. In stroke patients with dysphagia, the CSA of the non-hemiplegic anterior belly of the digastric muscle was smaller than that in stroke patients without dysphagia, whereas the geniohyoid CSA did not differ significantly between the groups [27]. Overall, these findings suggest that morphological alterations in swallowing-related muscles can be detected using quantitative ultrasonography after a stroke, although their directions and magnitudes vary across anatomical structures.

### 4.2. Alterations in Muscle Quality

Compared with studies of muscle quantity and morphology, considerably fewer studies have investigated muscle quality in patients with post-stroke dysphagia. Among the studies included in this review, only one evaluated an elastography-derived parameter, shear wave velocity (SWV), of the tongue muscle in patients with post-stroke dysphagia [41]. The tongue SWV did not differ significantly between patients with dysphagia and healthy controls. None of the included studies quantitatively evaluated the echo intensity, echogenicity, or texture-related features of the tongue or suprahyoid muscles. Consequently, the current evidence is insufficient to determine whether alterations in muscle quality contribute to swallowing dysfunction independently of changes in muscle size. Although changes in tissue composition, such as fatty infiltration and fibrosis, may influence muscle function, these characteristics have not been quantitatively evaluated in swallowing-related muscles after stroke in the studies included in this review. Therefore, muscle quality represents an important gap in the ultrasound assessment of post-stroke dysphagia and warrants further investigation using standardized imaging protocols.

### 4.3. Alterations in Dynamic Swallowing Function

Dynamic ultrasound studies have demonstrated alterations in both spatial/kinematic and temporal characteristics of swallowing-related movements in patients with stroke. Spatial and kinematic measures generally indicate reduced movement amplitudes of the tongue, suprahyoid muscles, and hyolaryngeal structures in patients with post-stroke dysphagia. Consistent with this overall pattern, tongue movement was reduced, as reflected by smaller changes in tongue thickness during swallowing and decreased radial displacement [34,37]. Similarly, reduced movement of the suprahyoid musculature has been demonstrated by lower geniohyoid radial displacement and reduced displacement of the suprahyoid muscle complex [35,36,37]. Additional suprahyoid muscle measures, including the suprahyoid muscle difference and ratio, decreased with greater dysphagia severity [35], while a lower suprahyoid muscle ratio was also observed in stroke patients with dysphagia compared with healthy controls [36]. Alterations in hyolaryngeal kinematics showed similar patterns. Reduced hyoid displacement has been observed in patients with dysphagia or aspiration [34,37,38], while a reduced hyoid–larynx approximation distance or ratio has been associated with post-stroke dysphagia and poorer swallowing function [25,37,39]. Collectively, these findings indicate that post-stroke dysphagia is characterized by reduced swallowing-related movements across multiple components of the swallowing mechanism.

Temporal and velocity measurements provided additional information on the speed and duration of swallowing-related movements. Prolonged movement of the tongue and geniohyoid muscles has been reported in patients with post-stroke dysphagia [37], whereas reduced tongue and geniohyoid movement speeds have been observed in patients with aspiration [38]. Temporal analysis of the suprahyoid muscle complex has also demonstrated prolonged swallowing-related movements, particularly in patients with severe dysphagia, although individual temporal parameters have not been consistently altered across studies [35,36]. These findings suggest that post-stroke dysphagia involves not only reductions in movement amplitude but also alterations in the timing and velocity of swallowing-related muscle movements. However, the evidence of asymmetry and coordination remains limited. Although bilateral ultrasonographic measurements have identified side-to-side differences in swallowing-related muscle morphology [27], specific symmetry indices have not yet been established. Moreover, despite assessments of spatial, velocity, and temporal characteristics, no study has directly quantified temporal or spatial coordination among multiple swallowing structures [35,36,37,38]. Thus, current ultrasound evidence provides only indirect information regarding asymmetry and coordination in post-stroke dysphagia. Overall, dynamic ultrasonography provides complementary information on the amplitude, velocity, and duration of swallowing-related movements; however, direct quantification of asymmetry and coordination remains limited.

### 4.4. Clinical Validation of Ultrasound Parameters

The clinical validity of ultrasound-derived parameters has been examined through comparisons with instrumental swallowing assessments, associations with clinical measures of swallowing function and severity, and evaluations of diagnostic or discriminative performance. Agreement with VFSS has been demonstrated in only a limited number of studies. Hsiao et al. compared hyoid displacement measured using submental ultrasonography with that obtained using VFSS and reported good agreement between the two methods [34]. Similarly, Huang et al. evaluated hyoid–larynx approximation using both ultrasonography and VFSS, supporting the validity of ultrasound in quantifying hyolaryngeal movement during swallowing [39]. These findings provide direct evidence that selected ultrasound-derived kinematic measures correspond to measurements obtained using an established instrumental swallowing assessment.

The associations between ultrasound-derived parameters and clinical swallowing function or dysphagia severity have been extensively examined. Several studies have compared ultrasound-derived morphological and dynamic parameters with functional oral intake, swallowing screening results, dysphagia severity, and aspiration status [25,27,34,35,36,37,38,39,41,42]. However, the findings were not consistent across parameters. Dynamic measures, including hyoid–larynx approximation and swallowing-related tongue, geniohyoid, and suprahyoid muscle movements, generally showed associations with impaired swallowing function or greater dysphagia severity, whereas some static morphological measures showed weaker or no associations. For example, sagittal tongue base thickness was not significantly associated with swallowing screening results in patients with acute ischemic stroke [42]. Thus, the clinical relevance of ultrasound parameters appears to depend on both the anatomical structure and type of parameter evaluated.

The diagnostic and discriminative performances of selected ultrasound parameters were also investigated. Hsiao et al. reported that a tongue thickness change of <1.0 cm identified tube-feeding-dependent dysphagia with a sensitivity of 70.0% and a specificity of 66.7%, whereas hyoid displacement <1.5 cm yielded a sensitivity of 73.3% and a specificity of 66.7%. When both criteria were required, the specificity increased to 93.3% [34]. Huang et al. similarly evaluated reduced hyoid–larynx approximation (<40% of the resting distance) as a criterion for identifying dysphagia [39]. More recent studies have extended this approach by examining combinations of dynamic ultrasound parameters to discriminate dysphagia severity from aspiration [35,37,38].

Current evidence suggests the potential clinical value of several ultrasound-derived parameters, particularly dynamic measures of swallowing biomechanics. However, direct validation against established instrumental assessments has been limited to a small number of studies and diagnostic thresholds have not been consistently replicated across independent populations. Further validation using standardized acquisition protocols, clearly defined clinical reference standards, and external cohorts is required before ultrasound-derived parameters can be used independently for clinical diagnosis or risk stratification.

## 5. Ultrasound Parameters for Rehabilitation Monitoring

Beyond characterizing swallowing impairments after stroke, ultrasonography can be used longitudinally to monitor treatment-related changes in the swallowing muscles and biomechanics throughout rehabilitation. In the five intervention studies included in this review, ultrasound parameters were selected according to the presumed therapeutic targets and included measures of muscle morphology, tongue morphology, mechanical properties, and hyolaryngeal movement [28,29,41,43,44]. These studies indicate that ultrasound can capture multiple dimensions of recovery, including structural adaptation and changes in swallowing-related movements, rather than providing only a cross-sectional description of impairment.

In exercise-based rehabilitation, ultrasound monitoring focuses primarily on the morphological adaptation of the suprahyoid musculature, together with changes in swallowing-related movements. Six weeks of jaw-opening or head-lift exercises increased the thickness of the mylohyoid and digastric muscles. These ultrasound-detected structural changes were accompanied by greater anterior and superior hyoid displacements on VFSS [43]. Similarly, an 8-week rehabilitation program incorporating proprioceptive neuromuscular facilitation or a chin tuck against resistance produced changes in suprahyoid muscle thickness, tongue root area, and hyolaryngeal elevation [29]. These ultrasound findings were accompanied by improvements in FEES-based swallowing safety, functional oral intake, dysphagia symptoms, and swallowing-related quality of life. Thus, repeated ultrasound measurements may document both morphological and kinematic responses to exercise-based rehabilitation.

Ultrasonography has also been used to monitor adjunctive swallowing interventions, with selected ultrasound parameters differing according to the presumed treatment mechanism. Kinesiology taping combined with repeated swallowing increased the thickness of the tongue, mylohyoid muscle, and anterior belly of the digastric muscle, demonstrating muscle-specific morphological responses to peripherally targeted intervention [28]. In swallowing therapy incorporating Iowa Oral Performance Instrument biofeedback or neuromuscular electrical stimulation, the affected-side masseter muscle thickness increased together with tongue strength and swallowing function. However, tongue thickness did not change significantly after swallowing therapy [41]. These contrasting findings indicate that functional improvement may not necessarily be accompanied by changes in every ultrasound parameter and that the responsiveness of ultrasound measures may differ according to the muscle or tissue properties being assessed.

For interventions targeting neural rather than muscular mechanisms, ultrasound monitoring primarily focuses on dynamic swallowing biomechanics. Following five sessions of intermittent theta burst stimulation combined with conventional therapy, the ultrasound-derived hyoid–larynx approximation ratio increased after real stimulation compared with that of sham stimulation [44]. This change occurred concurrently with improvements in VFSS-based Penetration–Aspiration Scale and functional dysphagia scale scores. These findings demonstrate the potential of ultrasound-derived kinematic measures for monitoring changes in hyolaryngeal movement associated with interventions that primarily target neural control rather than direct muscular adaptation.

Across the included studies, the ultrasound parameters used for rehabilitation monitoring generally corresponded to the therapeutic approach and the presumed target of treatment. Muscle thickness and area were predominantly used to monitor morphological adaptation during exercise-based or peripheral interventions, whereas hyoid–larynx approximation and hyolaryngeal elevation were used to capture dynamic biomechanical changes. Elastography-derived measures have also been explored as potential indicators of changes in tissue mechanical properties. However, their responsiveness to rehabilitation has not been consistently demonstrated. These findings extend the application of ultrasound from identifying post-stroke abnormalities to tracking structural and kinematic changes during recovery and highlight the importance of selecting ultrasound parameters that correspond to the specific component targeted by the rehabilitation intervention.

## 6. Clinical Applications and Future Directions

### 6.1. Clinical Applications

**Figure 1:**
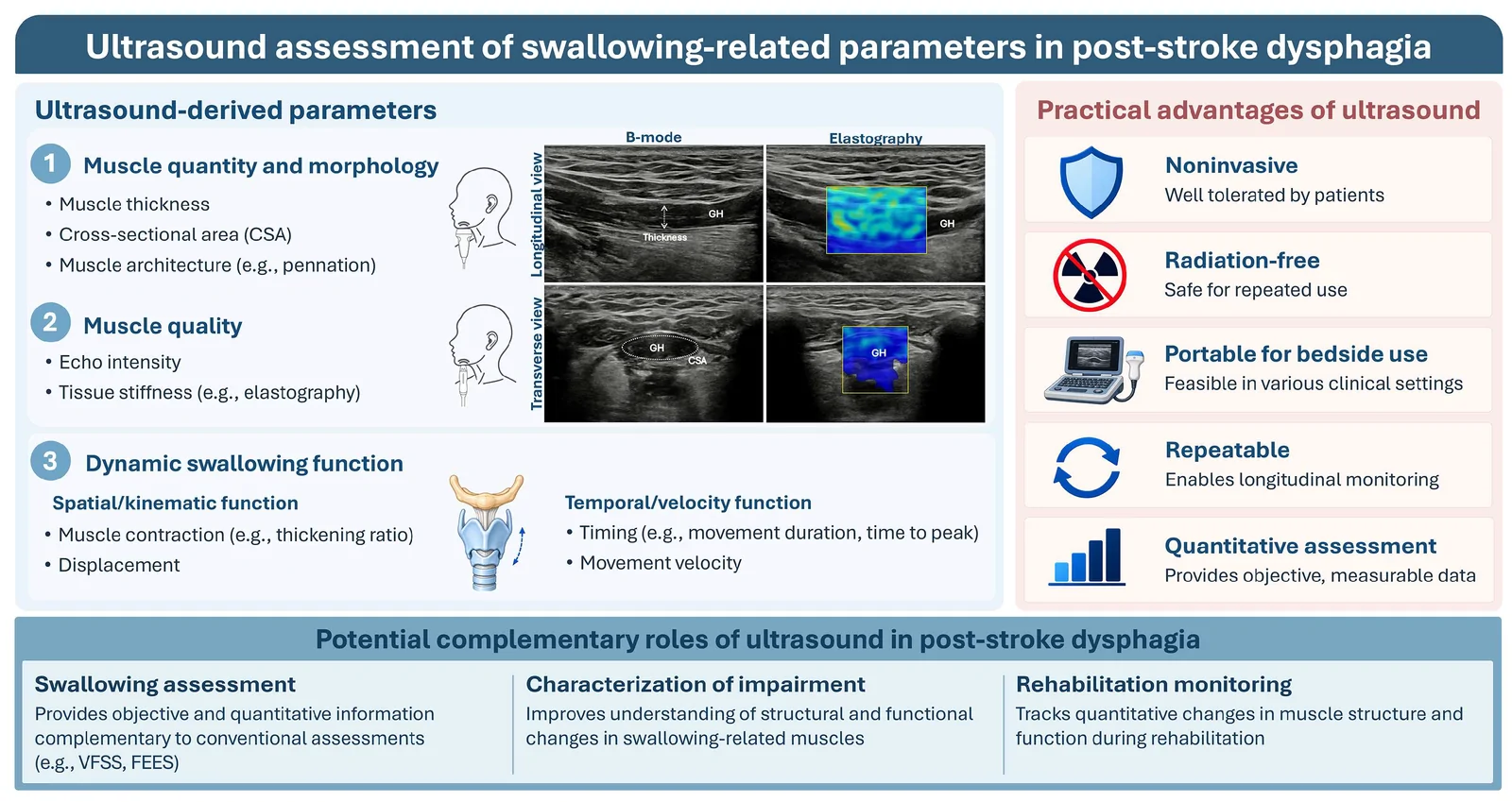
Ultrasound assessment of swallowing-related parameters in post-stroke dysphagia. Ultrasound-derived measures encompass muscle quantity and morphology, muscle quality, and dynamic swallowing function and may provide complementary information for swallowing assessment, characterization of impairment, and rehabilitation monitoring. FEES, fiberoptic endoscopic evaluation of swallowing; GH, geniohyoid muscle; VFSS, videofluoroscopic swallowing study.

Ultrasound may complement conventional assessment of post-stroke dysphagia by providing quantitative information on swallowing muscle morphology and movement (Figure 1). Its portability, lack of ionizing radiation, and suitability for repeated bedside assessment make it particularly useful for monitoring recovery [22,45,46]. Morphological measures, such as muscle thickness and cross-sectional area, may be selected when the clinical interest is muscle quantity or structural adaptation. In contrast, dynamic measures, including tongue and suprahyoid movement, hyoid excursion, and hyoid–larynx approximation, may be more appropriate for evaluating swallowing biomechanics. Given its relative ease of application and suitability for repeated bedside measurements, ultrasound may provide a practical complementary tool for initial and serial assessment of swallowing function. When a more comprehensive evaluation of swallowing safety and bolus flow is required, ultrasound can be used in conjunction with VFSS or FEES, allowing muscle- and movement-related ultrasound measures to complement direct assessment of bolus passage, penetration, and aspiration [19,47].

Ultrasound also has potential value in rehabilitation monitoring. Ultrasound parameters should be selected according to the presumed target of the intervention. Muscle thickness or area may be appropriate for interventions intended to induce muscular adaptation, whereas hyoid displacement, hyoid–larynx approximation, and hyolaryngeal elevation may be more responsive to interventions targeting swallowing movement or neuromotor control. However, clinical swallowing function may improve without corresponding changes in every ultrasound parameter, as demonstrated by improvements in tongue strength and swallowing function despite no significant change in tongue thickness [41]. Individual ultrasound parameters should therefore not be interpreted as isolated indicators of treatment effectiveness. Instead, morphological, mechanical, and kinematic measures should be interpreted alongside VFSS, FEES, tongue strength, functional oral intake, and patient-reported outcomes to identify potential discrepancies between physiological adaptation and functional improvement.

### 6.2. Current Limitations and Future Directions

The clinical application of swallowing ultrasound is currently limited by methodological heterogeneity across studies. Differences in probe placement, imaging plane, swallowing task, target structure, and outcome definition restrict direct comparison and may contribute to inconsistent findings. Moreover, reliability, responsiveness, and diagnostic thresholds have been established for only a limited number of ultrasound parameters. Standardized acquisition and analysis protocols, together with external validation against VFSS and FEES, are therefore needed before ultrasound measures can be consistently incorporated into clinical decision-making. Current evidence is also concentrated on muscle thickness and movement amplitude, with limited research on muscle quality, bilateral asymmetry, and coordination among swallowing structures. Future longitudinal studies should determine how these parameters change throughout stroke recovery and which measures are responsive to specific rehabilitation approaches. The development of muscle-quality measures, standardized bilateral symmetry indices, and time-synchronized, multi-structural assessments of tongue movement, suprahyoid muscle contraction, hyoid displacement, and laryngeal elevation may enable a more integrated evaluation of swallowing coordination beyond isolated measures of movement amplitude or duration. This review was also limited to searches of two databases without supplementary reference-list searching; therefore, some relevant studies may not have been identified.

## 7. Conclusions

Ultrasound can provide quantitative measures of swallowing-related muscle morphology and movement after stroke and may complement conventional swallowing assessments. Dynamic measures may be useful for characterizing swallowing impairment, while their role in monitoring rehabilitation-related changes requires further investigation. More consistent measurement methods and further validation are needed before ultrasound-derived measures can be routinely applied in clinical practice.

## Figures and Tables

**Table 1:** Characteristics of ultrasound studies for assessment and rehabilitation monitoring of post-stroke dysphagia.

Author(s) (Year)	Stroke Characteristics	Comparison/Intervention	Target Structure	US Mode/Image Acquisition	US Parameters	Reference Swallowing Assessment	Main Findings
Muscle quantity and morphology							
Li et al. (2025) [41]	Stroke within 1–12 months (mean 90.2 days)	Stroke patients with dysphagia vs. healthy controls **Intervention** ‡ Swallowing therapy with IOPI biofeedback or NMES	Tongue	Not specified/submental transverse scan in a relaxed state; probe type and frequency not specified	Tongue thickness	FOIS, IOPI (tongue strength and endurance)	Tongue thickness did not significantly differ between stroke patients with dysphagia and healthy controls and did not significantly change following swallowing therapy. Despite the absence of significant changes in tongue thickness, tongue strength and FOIS scores significantly improved following swallowing therapy.
Hsueh et al. (2024) [42]	Acute stroke within 2 weeks of onset	Stroke patients (with or without dysphagia) vs. healthy controls	Tongue	B-mode/submental sagittal scan at rest; 2–5 MHz convex probe	Tongue thickness	3-SSS ^†^	Stroke patients exhibited significantly greater STBT values than healthy controls. No significant association was found between ultrasound tongue parameters and the 3-SSS.
Ata and Gurcay (2026) [27]	Stroke onset to evaluation (days) - Dysphagia: 70 (37–206) - Non-dysphagia: 95 (42–196.5)	Stroke patients with dysphagia vs. without dysphagia	SHM	B-mode/submental transverse scan at rest; 5–12 MHz linear probe	Mylohyoid thickness; anterior digastric CSA; geniohyoid CSA	FOIS ^†^, EAT-10 ^†^	Stroke patients with dysphagia exhibited significantly lower mylohyoid muscle thickness and non-hemiplegic anterior digastric muscle CSA compared with those without dysphagia, whereas geniohyoid muscle CSA did not differ significantly. None of the ultrasound-derived swallowing muscle parameters independently predicted dysphagia.
Sung et al. (2026) [35]	Acute stroke within 5 days of onset	Stroke patients with mild dysphagia vs. severe dysphagia	SHM	B-mode/submental transverse scan during swallowing; 12-MHz linear probe	SHM complex thickness	VFSS (PAS, DOSS) ^†^	SHM thickness was significantly lower in patients with severe dysphagia than in those with mild dysphagia and was significantly correlated with VFSS-based PAS and DOSS scores.
Choi et al. (2020) [43]	Stroke within 1–5 months; with dysphagia	**Intervention** ^‡^ JOE vs. HLE	SHM	Not specified/submental scan between the hyoid bone and chin; 10-MHz linear and convex probe	Digastric and mylohyoid thickness	VFSS (hyoid movement)	Digastric and mylohyoid muscle thickness significantly increased after both JOE and HLE, with no significant between-group differences. VFSS-derived anterior and superior hyoid movement also significantly increased in both groups, with no significant between-group differences.
Jung et al. (2020) [28]	Stroke within 6 months; with dysphagia	**Intervention** ^‡^ KT vs. sham taping	Tongue; SHM	Not specified/probe placement not specified; 10-MHz linear and convex probe	Tongue thickness, mylohyoid, anterior belly of the digastric muscle thickness	None	Tongue, mylohyoid, and anterior digastric muscle thickness significantly increased after KT compared with those after sham taping.
Özcan et al. (2025) [29]	Stroke within 1.5–36 months; with dysphagia	**Intervention** ^‡^ PNF vs. CTAR vs. conventional therapy	Tongue; SHM	Not specified/submental transverse scan 10-MHz linear probe (for SHM); convex probe, frequency not specified (for tongue root)	SHM thickness, tongue CSA	FEES (PAS, NIH-SSS); FOIS; EAT-10; SWAL-QOL	SHM thickness and tongue root CSA improved after treatment, with greater improvements in the PNF and CTAR group than in the conventional exercise group. EAT-10, NIH-SSS, FOIS, and FEES-based PAS also significantly improved after treatment.
Muscle quality							
Li et al.(2025) [41]	Stroke within 1–12 months (mean 90.2 days)	Stroke patients with dysphagia vs. healthy controls **Intervention** ^‡^ Swallowing therapy with IOPI biofeedback or NMES	Tongue	Not specified/longitudinal scan along the muscle fibers in a relaxed state; linear probe, frequency not specified	SWV	FOIS, IOPI (tongue strength and endurance)	Tongue SWV did not significantly differ between stroke patients with dysphagia and healthy controls. Although tongue SWV was assessed before and after swallowing therapy, the pre–post results were not reported. Tongue strength and anterior tongue endurance were reduced in stroke patients with dysphagia, while tongue strength and FOIS scores significantly improved following swallowing therapy.
Spatial/kinematic function							
Hsiao et al. (2012) [34]	Stroke onset to evaluation (days) - Tube-feeding-dependent stroke: 69 ± 46 - Oral-intake stroke: 45 ± 24	Tube-feeding-dependent stroke patients vs. oral-intake stroke patients vs. healthy controls	Tongue; hyoid	B-mode/submental midsagittal scan during swallowing; 3.5-MHz convex probe	Tongue thickness change; hyoid displacement	VFSS (hyoid displacement) ^†^	Tube-feeding-dependent stroke patients showed significantly smaller tongue thickness change and hyoid displacement than oral-intake patients and healthy controls. Ultrasound-derived hyoid displacement showed good agreement with VFSS.
Huang et al. (2009) [39]	Stroke onset to evaluation (days) - Dysphagia: 59.0 ± 27.6 - Non-dysphagia: 45.2 ± 32.8	Stroke patients with dysphagia vs. without dysphagia vs. healthy controls	Hyoid–larynx	B-mode/anterior neck longitudinal scan at rest and during swallowing; 10-MHz convex probe	Hyoid–larynx approximation distance; approximation ratio	VFSS (Hyoid–larynx approximation distance; approximation ratio) ^†^	Dysphagic stroke patients showed significantly reduced approximation distance and approximation ratio. Ultrasound-derived approximation ratio was comparable to that measured by VFSS; an approximation ratio <40% identified dysphagia with 75.0% sensitivity and 77.1% specificity.
Picelli et al. (2021) [25]	Acute stroke within 1 week of onset	Stroke patients with dysphagia vs. without dysphagia	Hyoid–larynx	B-mode/anterior neck longitudinal scan at rest and during swallowing; 6-MHz linear probe	Hyoid–larynx approximation distance; approximation ratio	GUSS ^†^, FOIS ^†^	Approximation distance and approximation ratio were significantly reduced in dysphagic patients and positively correlated with GUSS and FOIS scores.
Shuai et al. (2025) [37]	Not reported	Stroke patients with dysphagia vs. healthy controls	Tongue; geniohyoid; hyoid–larynx	B- and M-mode/submental midsagittal scan during swallowing; 3.5-MHz convex probe	Tongue radial displacement; geniohyoid radial displacement; hyoid displacement; hyoid–larynx approximation distance	None	Stroke patients with dysphagia showed significantly reduced tongue and geniohyoid radial displacement, hyoid displacement, and hyoid–larynx approximation distance compared with healthy controls.
Zhan et al. (2026) [38]	Stroke within 3 months of onset	Stroke patients with dysphagia: aspiration vs. penetration vs. no penetration/aspiration	Hyoid	B-mode/submental midsagittal scan at rest and during swallowing; 1–6 MHz convex probe	Hyoid displacement; hyoid displacement/resting distance ratio	VFSS or FEES (PAS) ^†^	Hyoid displacement and hyoid displacement/resting distance ratio were significantly reduced in patients with aspiration and were negatively correlated with PAS scores.
Sung et al. (2024) [36]	Acute stroke	Stroke patients with dysphagia vs. healthy controls	SHM complex	M-mode/submental transverse scan during swallowing; 6–15 MHz linear probe	SHM displacement; SHM difference; SHM ratio	None	Stroke patients with dysphagia showed a significantly lower SHM ratio than healthy controls.
Sung et al. (2026) [35]	Acute stroke within 5 days of onset	Stroke patients with mild dysphagia vs. severe dysphagia	SHM complex	M-mode/submental transverse scan during swallowing; 12-MHz linear probe	SHM displacement; SHM difference; SHM ratio	VFSS (PAS, DOSS) ^†^	SHM displacement, difference, and ratio were significantly lower in patients with severe dysphagia compared with those with mild dysphagia. SHM displacement and difference were significantly correlated with VFSS-based PAS and DOSS scores.
Özcan et al. (2025) [29]	Stroke within 1.5–36 months, with dysphagia	**Intervention** ^‡^ PNF vs. CTAR vs. conventional therapy; pre–post intervention	Hyoid–larynx	Not specified/submental sagittal scan at rest and during swallowing; 10-MHz convex probe	Hyolaryngeal elevation	FEES (PAS, NIH-SSS); FOIS; EAT-10; SWAL-QOL	Hyolaryngeal elevation improved after treatment, with greater improvements in the PNF and CTAR groups than in the conventional exercise group. EAT-10, NIH-SSS, FOIS, and FEES-based PAS also significantly improved after treatment.
Suh et al. (2024) [44]	More than 2 weeks post-stroke; with dysphagia	**Intervention** ^‡^ Real iTBS vs. sham iTBS; pre–post intervention	Hyoid–larynx	Not specified/anterior neck midline longitudinal scan at rest and during swallowing; convex probe	Resting distance; shortest distance; hyoid–larynx approximation distance; approximation ratio	VFSS (PAS, FDS)	Hyoid–larynx approximation distance and approximation ratio significantly increased after real iTBS compared with sham. VFSS-based PAS and FDS also showed significantly greater improvements in the real iTBS group than in the sham group.
Temporal/velocity function							
Shuai et al. (2025) [37]	Not reported	Stroke patients with dysphagia vs. healthy controls	Tongue; geniohyoid	M-mode/submental midsagittal scan during swallowing; 3.5-MHz convex probe	Tongue movement duration; geniohyoid movement duration	None	Tongue and geniohyoid movement durations were significantly prolonged in stroke patients with dysphagia compared with healthy controls.
Zhan et al. (2026) [38]	Stroke within 3 months of onset	Stroke patients with dysphagia: aspiration vs. penetration vs. no penetration/aspiration	Tongue; geniohyoid	M-mode/submental midsagittal scan at rest and during swallowing; 1–6 MHz convex probe	Tongue movement speed; geniohyoid movement speed	VFSS or FEES (PAS) ^†^	Tongue and geniohyoid movement speeds decreased with increasing PAS score and were negatively correlated with PAS scores.
Sung et al. (2024) [36]	Acute stroke	Stroke patients with dysphagia vs. healthy controls	SHM complex	M-mode/submental transverse scan during swallowing; 6–15 MHz linear probe	Peak-to-peak time; total duration	None	Total duration was significantly prolonged in stroke patients with dysphagia compared with healthy controls, whereas peak-to-peak time did not differ significantly.
Sung et al. (2026) [35]	Acute stroke within 5 days of onset	Stroke patients with mild dysphagia vs. severe dysphagia	SHM complex	M-mode/submental transverse scan during swallowing; 12-MHz linear probe	Peak-to-peak time; total duration	VFSS (PAS, DOSS) ^†^	Peak-to-peak time and total duration were significantly prolonged in patients with severe dysphagia compared with those with mild dysphagia. Peak-to-peak time was negatively correlated with DOSS, while total duration was positively correlated with PAS and negatively correlated with DOSS.

Abbreviations: 3-SSS, 3-step swallowing test; CSA, cross-sectional area; CTAR, chin tuck against resistance; DOSS, Dysphagia Outcome and Severity Scale; EAT-10, Eating Assessment Tool-10; FDS, Functional Dysphagia Scale; FEES, fiberoptic endoscopic evaluation of swallowing; FOIS, Functional Oral Intake Scale; GUSS, Gugging Swallowing Screen; HLE, head-lift exercise; IOPI, Iowa Oral Performance Instrument; iTBS, intermittent theta-burst stimulation; JOE, jaw-opening exercise; KT, kinesiology taping; NIH-SSS, National Institutes of Health Swallowing Safety Scale; NMES, neuromuscular electrical stimulation; PAS, Penetration–Aspiration Scale; PNF, proprioceptive neuromuscular facilitation; SHM, suprahyoid muscle; STBT, sagittal tongue base thickness; SWAL-QOL, Swallowing Quality of Life; SWV, shear-wave velocity; US, ultrasonography; VFSS, videofluoroscopic swallowing study. Values are presented as mean ± standard deviation or median (interquartile range), as reported in the original studies. ^†^ The relationship between ultrasound parameters and swallowing assessment was statistically examined. ^‡^ Studies using ultrasound for rehabilitation monitoring.

## Data Availability

No new data were created or analyzed in this study. All the information included in this review was obtained from previously published studies.

## Abbreviations

The following abbreviations are used in this manuscript:

3-SSS	3-step swallowing test
B-mode	Brightness mode
CSA	Cross-sectional area
CTAR	Chin tuck against resistance
DOSS	Dysphagia Outcome and Severity Scale
EAT-10	Eating Assessment Tool-10
FDS	Functional Dysphagia Scale
FEES	Fiberoptic endoscopic evaluation of swallowing
FOIS	Functional Oral Intake Scale
GUSS	Gugging Swallowing Screen
HLE	Head-lift exercise
IOPI	Iowa Oral Performance Instrument
iTBS	Intermittent theta-burst stimulation
JOE	Jaw-opening exercise
KT	Kinesiology taping
M-mode	Motion mode
NIH-SSS	National Institutes of Health Swallowing Safety Scale
NMES	Neuromuscular electrical stimulation
PAS	Penetration–Aspiration Scale
PNF	Proprioceptive neuromuscular facilitation
SHM	Suprahyoid muscle
STBT	Sagittal tongue base thickness
SWAL-QOL	Swallowing Quality of Life
SWV	Shear-wave velocity
US	Ultrasonography
VFSS	Videofluoroscopic swallowing study

## References

[1] Cohen D.L., Roffe C., Beavan J., Blackett B., Fairfield C.A., Hamdy S., Havard D., McFarlane M., McLauglin C., Randall M. (2016). Post-stroke dysphagia: A review and design considerations for future trials. Int. J. Stroke.

[2] Dziewas R., Michou E., Trapl-Grundschober M., Lal A., Arsava E.M., Bath P.M., Clavé P., Glahn J., Hamdy S., Pownall S. (2021). European Stroke Organisation and European Society for Swallowing Disorders guideline for the diagnosis and treatment of post-stroke dysphagia. Eur. Stroke J..

[3] Cheng I., Hamdy S. (2022). Metaplasticity in the human swallowing system: Clinical implications for dysphagia rehabilitation. Neurol. Sci..

[4] Huckabee M.L., Flynn R., Mills M. (2023). Expanding Rehabilitation Options for Dysphagia: Skill-Based Swallowing Training. Dysphagia.

[5] Boaden E., Burnell J., Hives L., Dey P., Clegg A., Lyons M.W., Lightbody C.E., Hurley M.A., Roddam H., McInnes E. (2021). Screening for aspiration risk associated with dysphagia in acute stroke. Cochrane Database Syst. Rev..

[6] Coelho P.M., Almeida P.L., Carmezim I., Silva A., Evangelista R., Dinis C., Martins T., Torres A., Gomes A., Caldas J. (2024). Mortality and Pulmonary Complications of Post-stroke Dysphagia: A Casuistic Review of an Acute Stroke Unit. Cureus.

[7] Smaoui S., Peladeau-Pigeon M., Steele C.M. (2022). Determining the Relationship Between Hyoid Bone Kinematics and Airway Protection in Swallowing. J. Speech Lang. Hear. Res..

[8] Kendall K.A. (2017). Evaluation of airway protection: Quantitative timing measures versus penetration/aspiration score. Laryngoscope.

[9] Allen J.E., Clunie G.M., Slinger C., Haines J., Mossey-Gaston C., Zaga C.J., Scott B., Wallace S., Govender R. (2021). Utility of ultrasound in the assessment of swallowing and laryngeal function: A rapid review and critical appraisal of the literature. Int. J. Lang. Commun. Disord..

[10] Potente P., Buoite Stella A., Vidotto M., Passerini M., Furlanis G., Naccarato M., Manganotti P. (2023). Application of Ultrasonography in Neurogenic Dysphagia: A Systematic Review. Dysphagia.

[11] Jones C.A., Colletti C.M., Ding M.C. (2020). Post-stroke Dysphagia: Recent Insights and Unanswered Questions. Curr. Neurol. Neurosci. Rep..

[12] Sasegbon A., Cheng I., Hamdy S. (2025). The neurorehabilitation of post-stroke dysphagia: Physiology and pathophysiology. J. Physiol..

[13] Steele C.M., Miller A.J. (2010). Sensory input pathways and mechanisms in swallowing: A review. Dysphagia.

[14] Hori K., Ono T., Iwata H., Nokubi T., Kumakura I. (2005). Tongue pressure against hard palate during swallowing in post-stroke patients. Gerodontology.

[15] Mann G., Hankey G.J. (2001). Initial clinical and demographic predictors of swallowing impairment following acute stroke. Dysphagia.

[16] Feng X., Todd T., Lintzenich C.R., Ding J., Carr J.J., Ge Y., Browne J.D., Kritchevsky S.B., Butler S.G. (2013). Aging-related geniohyoid muscle atrophy is related to aspiration status in healthy older adults. J. Gerontol. A Biol. Sci. Med. Sci..

[17] Fujishima I., Fujiu-Kurachi M., Arai H., Hyodo M., Kagaya H., Maeda K., Mori T., Nishioka S., Oshima F., Ogawa S. (2019). Sarcopenia and dysphagia: Position paper by four professional organizations. Geriatr. Gerontol. Int..

[18] Huckabee M.L., Macrae P., Lamvik K. (2015). Expanding Instrumental Options for Dysphagia Diagnosis and Research: Ultrasound and Manometry. Folia Phoniatr. Logop..

[19] Allen J.E., Clunie G.M., Winiker K. (2021). Ultrasound: An emerging modality for the dysphagia assessment toolkit?. Curr. Opin. Otolaryngol. Head Neck Surg..

[20] Hsiao M.Y., Wu C.H., Wang T.G. (2021). Emerging Role of Ultrasound in Dysphagia Assessment and Intervention: A Narrative Review. Front. Rehabil. Sci..

[21] Song W., Wu M., Wang H., Pang R., Zhu L. (2024). Prevalence, risk factors, and outcomes of dysphagia after stroke: A systematic review and meta-analysis. Front. Neurol..

[22] Wang Y., Wang Q., Yu W., Cai J., Shan L., Wang X., Fang M., Lu X. (2025). Validity and Reliability of Ultrasound Assessment of Swallowing Function: A Systematic Review and Meta-Analysis. J. Oral. Rehabil..

[23] Maeda K., Nagasaka M., Nagano A., Nagami S., Hashimoto K., Kamiya M., Masuda Y., Ozaki K., Kawamura K. (2023). Ultrasonography for Eating and Swallowing Assessment: A Narrative Review of Integrated Insights for Noninvasive Clinical Practice. Nutrients.

[24] Van den Broeck J., Héréus S., Cattrysse E., Raeymaekers H., Scafoglieri A. (2025). Ultrasound cut-off values for muscle thickness, cross-sectional area, and echo intensity in Caucasian adults: A narrative review. Quant. Imaging Med. Surg..

[25] Picelli A., Modenese A., Poletto E., Businaro V., Varalta V., Gandolfi M., Bonetti B., Smania N. (2021). May ultrasonography be considered a useful tool for bedside screening of dysphagia in patients with acute stroke? A cohort study. Minerva Med..

[26] de Andrade R.A., Pernambuco L.A., de Almeida A.N.S., Mulatinho M., Dos Santos E.N.F., da Silva H.J. (2025). Methodological Procedures to Acquire and Analyze Ultrasound Images of Swallowing: A Scoping Review. Dysphagia.

[27] Ata A.M., Gurcay E. (2026). Relationship Between Swallowing Muscle Morphology and Dysphagia in Patients Followed With Stroke. J. Oral. Rehabil..

[28] Jung Y.J., Kim H.J., Choi J.B., Park J.S., Hwang N.K. (2020). Effect of Dysphagia Rehabilitation Using Kinesiology Taping on Oropharyngeal Muscle Hypertrophy in Post-Stroke Patients: A Double Blind Randomized Placebo-Controlled Trial. Healthcare.

[29] Özcan E.N., Ünlüer N., Öztürk M., Erdur Ö. (2025). Does Proprioceptive Neuromuscular Facilitation Approach Have an Effect on Swallowing Function, Muscle Morphology and Quality of Life in Dysphagic Stroke Patients? A Randomised Controlled Trial. J. Oral. Rehabil..

[30] Safer V.B., Catikkas N.M., Gultekin A., Safer U. (2026). Ultrasonographic Geniohyoid Muscle Measurements and Their Relationship With Quality of Life and Functional Outcomes in Older Patients With Stroke. J. Clin. Ultrasound.

[31] Pillen S., Tak R.O., Zwarts M.J., Lammens M.M., Verrijp K.N., Arts I.M., van der Laak J.A., Hoogerbrugge P.M., van Engelen B.G., Verrips A. (2009). Skeletal muscle ultrasound: Correlation between fibrous tissue and echo intensity. Ultrasound Med. Biol..

[32] Ticinesi A., Meschi T., Narici M.V., Lauretani F., Maggio M. (2017). Muscle Ultrasound and Sarcopenia in Older Individuals: A Clinical Perspective. J. Am. Med. Dir. Assoc..

[33] Ogawa N., Mori T., Fujishima I., Wakabayashi H., Itoda M., Kunieda K., Shigematsu T., Nishioka S., Tohara H., Yamada M. (2018). Ultrasonography to Measure Swallowing Muscle Mass and Quality in Older Patients With Sarcopenic Dysphagia. J. Am. Med. Dir. Assoc..

[34] Hsiao M.Y., Chang Y.C., Chen W.S., Chang H.Y., Wang T.G. (2012). Application of ultrasonography in assessing oropharyngeal dysphagia in stroke patients. Ultrasound Med. Biol..

[35] Sung J.H., Lee S.H., Sohn J.H., Han S.W., Choi H., Shin S.H., Lee J.J., Kim C. (2026). Dynamic suprahyoid muscle ultrasound in acute stroke: A prospective observational study for developing a dysphagia severity score. Eur. J. Phys. Rehabil. Med..

[36] Sung J.H., Baek S.H., Park J.W., Lee J.H., Son M.H., Kim B.J. (2024). Dynamic suprahyoid muscle ultrasound in assessing oropharyngeal dysphagia in neurological disorders. Eur. J. Phys. Rehabil. Med..

[37] Shuai J., Pian L., Tian L., Wang L., Deng M., Cheng C. (2025). Application of B+M-Mode Ultrasound in Evaluating Dysphagia in Elderly Stroke Patients. Ultrasound Med. Biol..

[38] Zhan S.Y., Wei L.M., Lin Z.C., Shao M.M. (2026). Association of tongue-hyoid bone motion parameters measured dynamically by submental ultrasound with the severity of post-stroke dysphagia. Clin. Biomech..

[39] Huang Y.L., Hsieh S.F., Chang Y.C., Chen H.C., Wang T.G. (2009). Ultrasonographic evaluation of hyoid-larynx approximation in dysphagic stroke patients. Ultrasound Med. Biol..

[40] Winstein C.J., Stein J., Arena R., Bates B., Cherney L.R., Cramer S.C., Deruyter F., Eng J.J., Fisher B., Harvey R.L. (2016). Guidelines for Adult Stroke Rehabilitation and Recovery: A Guideline for Healthcare Professionals From the American Heart Association/American Stroke Association. Stroke.

[41] Li G.Y., Huang Y.C., Wu J.Y., Leong C.P., Cheng C.H. (2025). Swallowing Therapy Effects on Oral Muscle Mass, Tongue Function, Swallowing, and Nutrition in Stroke Patients with Dysphagia. NeuroRehabilitation.

[42] Hsueh S.J., Hsu B., Chang K.C. (2024). The role of the tongue in post-stroke dysphagia and obstructive sleep apnea: Correlation with sonography measurement. Clin. Neurol. Neurosurg..

[43] Choi J.B., Jung Y.J., Park J.S. (2020). Comparison of 2 types of therapeutic exercise: Jaw opening exercise and head lift exercise for dysphagic stroke: A pilot study. Medicine.

[44] Suh I., You J., Son S., Bae J.S., Lim J.Y. (2024). The effect of real versus sham intermittent theta burst transcranial magnetic stimulation combined with conventional treatment on poststroke dysphagia: A randomized controlled trial. Int. J. Rehabil. Res..

[45] Winiker K., Hammond R., Thomas P., Dimmock A., Huckabee M.L. (2022). Swallowing assessment in patients with dysphagia: Validity and reliability of a pocket-sized ultrasound system. Int. J. Lang. Commun. Disord..

[46] Barron K., Blaivas M., Blaivas L., Sadler J., Deal I. (2024). Bedside Ultrasound to Identify and Predict Severity of Dysphagia Following Ischemic Stroke: Human Versus Artificial Intelligence. Ultrasound Med. Biol..

[47] Espitalier F., Fanous A., Aviv J., Bassiouny S., Desuter G., Nerurkar N., Postma G., Crevier-Buchman L. (2018). International consensus (ICON) on assessment of oropharyngeal dysphagia. Eur. Ann. Otorhinolaryngol. Head Neck Dis..

